# Association Between Blood Urea Nitrogen/Albumin and the Incidence as Well as Progression of Type 2 Diabetes

**DOI:** 10.3390/nu17010113

**Published:** 2024-12-30

**Authors:** Yongqi Liang, Rui Zhou, Chenxi Jin, Jingjing Liang, Xiaoyan Wang, Weidong Fan, Xianbo Wu, Mengchen Zou

**Affiliations:** 1 Department of Epidemiology, School of Public Health, Southern Medical University, Guangzhou 510515, China; lyq5151@i.smu.edu.cn (Y.L.); ray_chou1996@i.smu.edu.cn (R.Z.); 3187042019@i.smu.edu.cn (W.F.); 2 Department of Epidemiology, School of Public Health, Guangdong Pharmaceutical University, Guangzhou 510310, China; 3 Department of Endocrinology and Metabolism, Nanfang Hospital, Southern Medical University, Guangzhou 510510, China; 18569065385@163.com (C.J.); ljj22320720@smu.edu.cn (J.L.); 1180011086@i.smu.edu.cn (X.W.)

**Keywords:** blood urea nitrogen/albumin, type 2 diabetes, macrovascular complications, microvascular complications, mortality

## Abstract

Background: An increased risk of multiple secondary diseases has been observed in individuals with diabetes, which contributes to the growing economic burden. Few studies have established the connection of blood urea nitrogen/albumin (BAR) with diabetes, and its link to subsequent diabetic complications and mortality remains unclear. We aimed to explore the association of BAR with the onset of type 2 diabetes mellitus (T2DM) and its dynamic progression. Methods: A total of 20,039 prediabetic participants aged 40–70, without diabetes or related events at baseline, were selected from the UK Biobank. We used a multistate regression model to assess the relationships between BAR and the trajectories of T2DM progression—from pre-T2DM to T2DM, complications, and ultimately mortality. Akaike information criterion (AIC), area under the curve (AUC), and *C-*statistic analyses were performed to compare the diagnostic performance of BAR with that of HbA1c for assessing T2DM progressions risk. Results: Over a mean 13-year follow-up, 5621 incident T2DM cases were identified, and among them, 1295 developed diabetes macrovascular complications, 574 developed diabetes microvascular complications, and 1264 died. BAR was significantly associated with the increased risk of T2DM (HR: 1.05, 95% CI: 1.02, 1.09), subsequent macrovascular complications (HR: 1.18, 95% CI: 1.12, 1.25), microvascular complications (HR 1.18, 95% CI: 1.08, 1.28), and further death (HR 1.18, 95% CI: 1.00, 1.39). The ability of BAR (AUC: 0.938, *C-*statistic: 0.739, *p* < 0.001) to predict diabetes progression was inferior to that of hemoglobin A1c (HbA1c) (AUC: 0.949, *C-*statistic: 0.809, *p* < 0.001). Conclusions: Although BAR is significantly positively correlated with the risk of progression at different stages of diabetes, its predictive performance is inferior to that of HbA1c and comparable to that of BUN and albumin. BAR does not demonstrate sufficient clinical significance in predicting diabetes progression, highlighting the need for further investigation.

## 1. Introduction

Advancements in longevity have heightened the likelihood of individuals experiencing the onset of two or more diseases, that is, multimorbidity [1]. Type 2 diabetes mellitus (T2DM) manifests with a variety of complications and frequently co-occurs with multiple microvascular and macrovascular complications [2]. Macrovascular complications, which encompass stroke, heart disease, cardiomyopathy, heart failure, and atrial fibrillation, contribute to nearly a third of global fatalities [3]; they account for approximately 95% of all cardiovascular disease (CVD) deaths [4], with CVD representing the main cause of illness and death in those with T2DM [5]. Microvascular complications, including retinopathy, neuropathy, and nephropathy, also pose significant health challenges. Diabetic retinopathy and nephropathy affect nearly 30–40% of patients with diabetes [6,7], while neuropathy may develop in approximately 50% of diabetic patients [8]. For individuals with diabetes, an extended life expectancy translates to a prolonged coexistence with multiple complications, which imposes a substantial economic burden on patients grappling with diabetic diseases [9,10,11].

Blood urea nitrogen (BUN) serves as a pivotal marker for metabolic disorders and various diseases; it is used as an indicator of the intricate interplay among nutritional status, protein metabolism, and renal function [12,13,14]. Strong experimental evidence indicates that BUN is proposed to be a key factor in reduced insulin sensitivity and impaired insulin secretion [15,16]. However, aging, gastrointestinal bleeding, corticosteroid use, and protein-rich diets can all contribute to elevated BUN levels by increasing urea production, making it difficult to rely on BUN measurements alone for precise predictions [17]. Albumin is extensively used as a biomarker in clinical applications as well due to its essential roles in maintaining osmotic balance, antioxidative activity, anti-inflammatory effects, and endothelial stability; its levels are affected by factors such as nutritional status, liver function, and catabolic activity [17]. Therefore, both BUN and albumin have intrinsic limitations when used independently in prognostication. The BUN to albumin ratio (BAR) has gained attention as a potential predictive marker in a range of diseases, particularly respiratory and septic conditions [18,19]. Additionally, most studies on BAR have focused on acute conditions (e.g., bleeding, dehydration, and heart failure) where elevated BAR is likely due to renal reabsorption from decreased circulating volume [20,21,22]. However, given the varied factors associated with elevated BUN and low albumin levels, BAR is considered to play a role in chronic disease development [23].

It remains unclear whether BAR affects macrovascular complications and subsequent mortality independently of other clinical and laboratory factors. The need for robust evidence, particularly from large-scale population studies, remains paramount to unravelling the nuanced relationship between BAR levels and cardiovascular risks. Although BAR has long been linked to mortality [24,25,26], limited studies have explored its association with complications and all-cause mortality in the diabetic patients. This remains a controversial topic requiring further prospective cohort studies. Notably, most research has focused on single complications [27,28], and the potential link between BAR and the progression of T2DM, from pre-T2DM to incident T2DM, complications, and death, remains unexplored. Based on previous studies, we can hypothesize that high BAR levels may increase the risk of various stages of T2DM progression.

Advanced statistical models, such as multistate models, allow for examining transitions between disease states and quantifying progression rates while accounting for concurrent complications [29]. To fill this knowledge gap, we aimed to examine the link between BAR and various stages of T2DM progression by utilizing a multistate model. In addition, we aimed to predict transition probabilities between stages for participants with different BAR levels. The insights derived from this statistical model will contribute valuable evidence regarding prioritizing actions for the optimization of prevention and management strategies for different stages of T2DM.

We aimed to predict transition probabilities between stages for participants with different BAR levels.

## 2. Methods

### 2.1. Study Population

The UK Biobank (UKB) is a vast biomedical database containing data from around 500,000 individuals aged 37–73, recruited across the UK between 2006 and 2010. All participants completed an extensive and validated questionnaire to gather information on sociodemographic variables, diet, and behavior. The UKB details have been outlined elsewhere [30]. Prediabetes was diagnosed following the 2021 guidelines set by the American Diabetes Association, which defines it by the presence of impaired fasting glucose, hemoglobin A1c (HbA1c) levels between 5.7% and 6.4% (39–47 mmol/mol), or both. In this analysis, we excluded participants without prediabetes (*n* = 472,725), with missing exposure data (*n* = 33), with experiences of diabetes-related events before T2DM diagnosis (*n* = 7676), with diabetes and diabetes-related events (*n* = 1963), and with missing follow-up data (*n* = 71), which resulted in a final sample of 20,039 (Figure S1). The UKB was approved by the North West Multicenter Research Ethics Committee, with all participants giving written informed consent at enrollment.

### 2.2. Exposure and Experimental Assessment

BAR is calculated as the ratio of BUN (mg/L) to albumin(g/L). BUN, albumin, and other blood biochemistries were measured using 10 immunoassay analyzers (4 Beckman Coulter DXI 800 and 6 DiaSorin Liaison XL) and 4 clinical chemistry analyzers (2 Siemens Advia 180 and 2 Beckman Coulter AU58000). These analyzers are manufactured by Beckman Coulter (Brea, CA, USA), DiaSorin (Saluggia, Vercelli, Italy), and Siemens (Erlangen, Germany), and are typically purchased through their official websites or authorized distributors. Through calculation using the formula of urea [mmol/L] × 1 = BUN [mmol/L], we established that 1 mmol/L urea is equivalent to 1 mmol/L of BUN, 1 BUN [mmol/L] = 28 BUN [mg/L]. For detailed conversion formulas, please refer to (https://unitslab.com/node/98#google_vignette, accessed on 30 November 2024). Specific details regarding the equipment and materials used in the measurement of each biomarker were obtained from resources available at (https://biobank.ctsu.ox.ac.uk/crystal/label.cgi?id=17518, accessed on 30 November 2024) and (https://biobank.ctsu.ox.ac.uk/crystal/label.cgi?id=100081, accessed on 30 November 2024) during the initial assessment. Rigorous quality control (QC) procedures, including the correction for technical outliers, were conducted by the UKB. Comprehensive information on blood analytes and counts, along with QC processes, can be found at (https://biobank.ctsu.ox.ac.uk/crystal/crystal/docs/serum_biochemistry.pdf, accessed on 30 November 2024) and (https://biobank.ctsu.ox.ac.uk/crystal/crystal/docs/haematology.pdf, accessed on 30 November 2024).

### 2.3. Ascertainment of Outcomes

The primary outcomes included the incidence of T2DM, subsequent complications, and all-cause mortality from follow-up to 1 March 2022. The diagnoses of T2DM and complications were mapped to a three-digit code of the International Classification of Disease (ICD-10). Data on UKB participants were obtained from NHS England for those in England and Wales, and from the NHS Central Register. Both providers currently provide extracts of death data on a quarterly basis. The follow-up period spanned from enrollment in the UKB study until the outcomes of interest, all-cause mortality, or the end of follow-up, whichever came first. All-cause and cause-specific mortality data were sourced from the national death registry, with specific causes including cancer, CVD, and diabetic complications, and they accounted for 58.80%,10.98%, and 1.09% of the overall deaths in our study, respectively. Table S1 provides details on the codes and data fields for the key outcomes.

### 2.4. Covariates

Previous studies demonstrated the association of the following covariates, which were selected for our analyses, with BAR of diabetic patients [31]: age, sex (male/female), (white/nonwhite), smoking status (never/current/former), drinking status (never/current/former), obese (body mass index > 30 kg/m^2^), physical activity level [32] (high, moderate, low), healthy diet score [32] (assessed using four components, red meat, fish, vegetable, and fruit intakes; a healthy diet had a diet score ≥ 3), chronic disease history (hypertension, hypercholesterolemia, and cancer), and family history of diabetes. Comorbidities were assessed using self-reported data, medication history, and hospital inpatient records. Table S1 presents the detailed information.

### 2.5. Statistical Analysis

Our dataset included a large sample with a normal distribution. Continuous data are expressed as mean (SD), and categorical data as frequencies (N) and percentages (%). One-way ANOVA and χ^2^ tests were used to assess differences among multiple groups for continuous and categorical variables, respectively.

Using a multistate cox regression model, we computed the hazard ratio (HR) and the 95% confidence interval (CI) to assess the associations between BAR and T2DM progression, from pre-T2DM to T2DM, complications, and all-cause mortality. The multistate model, an extension of the competing risk model, assesses the impact of exposures on various stages of disease progression [33] and has been used to study risk factors for cardiometabolic multimorbidity and diabetic complications [34,35].

**Figure 1:**
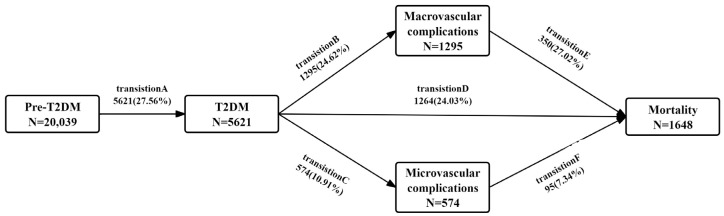
Transitions from pre-T2DM to T2DM, diabetic complications, and all-cause death. Abbreviations: T2DM, type 2 diabetes mellitus.

In this study, we considered six transition phases based on the natural progression of T2DM (Figure 1): (A) pre-T2DM to T2DM (*n* = 5621); (B) T2DM to any of the macrovascular complications (*n* = 1295); (C) T2DM to any of the microvascular complications (*n* = 574); (D) T2DM to all-cause death (*n* = 1264); (E) diabetic macrovascular complication to all-cause mortality (*n* = 350); and (F) diabetic microvascular complication to all-cause mortality (*n* = 95). In certain instances, for participants who experienced multiple states on the same date, we calculated the entry date for the first state by subtracting 0.5 [36]. Three models were developed to examine the associations between BAR and the six T2DM trajectory transitions. The unadjusted model was calculated first, followed by adjustments for age and sex (Model 1). Model 2 included additional adjustments for drinking, obesity, smoking, diet, physical activity, eGFR, family history of diabetes, and histories of high cholesterol, hypertension, and cancer. Next, Model 2 was used as the primary model for subsequent analyses to minimize potential bias. We also applied restricted cubic splines (RCS) to assess nonlinearity in the BAR–T2DM trajectory association, using likelihood ratio tests to examine nonlinear relationships.

Cumulative probabilities of transitioning to the later state from the prior state were predicted during follow-up for participants with varying BAR levels. For participants at aged 60 years old, cumulative predicted probabilities were calculated based on Model 2.

The sensitivity analysis examined the relationship between BAR and various stages of the T2DM trajectory, excluding participants who developed cancer during follow-up, those with extreme BAR values (above the 99th percentile or below the 1st percentile), those with a follow-up duration of less than two years, and those patients with both macrovascular and microvascular complications. To investigate the influence of BAR on T2DM progression while mitigating the effect of low estimated glomerular filtration rate (eGFR) levels [28], participants with an eGFR below 60 mL/min/1.73 m^2^ were excluded from our analysis. We also investigated associations between BAR and risk of four progressions of T2DM (patients with diabetic pan-vascular disease). Subgroup analyses were conducted based on age (<60 or ≥60 years), sex (male/female), race (white/nonwhite), obesity (yes/no), hyperlipemia (yes/no), hypertension (yes/no), healthy diet (yes/no), and physical activity (high/moderate/low). Interaction terms between the layered factors and T2DM trajectory stages were included in multivariate Cox regression models (based on model 2) to calculate *p*-values using likelihood ratio tests, comparing models with and without the interaction terms.

Akaike information criterion (AIC), area under the curve (AUC), and *C-*statistic analyses were performed to compare the diagnostic performance of BAR with that of hemoglobin A1c (HbA1c) for assessing T2DM progressions risk.

Data processing and analysis were conducted using R version 4.3.0. All statistical tests were two-sided, with significance set at *p* < 0.05.

## 3. Results

### 3.1. Characteristics at Baseline

This study included 20,039 eligible participants (mean [SD] age: 58.01 ± 7.66 years, 53.92% were females). Table 1 shows the baseline characteristics of participants grouped by quartiles of BAR exposure. Participants with a high exposure to BAR were older and likely to be white. Over an average follow-up period of 13.12 years (SD = 0.83), 5621 (5.14/1000 person years), participants experienced T2DM. Among these participants, 1295 (20.15/1000 person years) developed diabetic macrovascular complications, 574 (8.48/1000 person years) developed diabetic microvascular complications, and 1264 (11.41/1000 person years) died without experiencing macrovascular complications. Among 1295 individuals with diabetic macrovascular complications, 350 died, resulting in a crude mortality rate of 22.54 per 1000 person-years. Of the 574 individuals with diabetic microvascular complications, 95 died, with a crude mortality rate of 13.36 per 1000 person-years. (Figure 1). The mean exposures to BAR in the six stages were 3.45, 3.51, 3.72, 3.82, 3.90, and 4.54 mg/g (Table S2). Participants included in the study were older, and were more likely to be female and white, compared to those excluded (Table S3).

### 3.2. Association Between BAR and Different Progressions of Type 2 Diabetes

**Figure 2:**
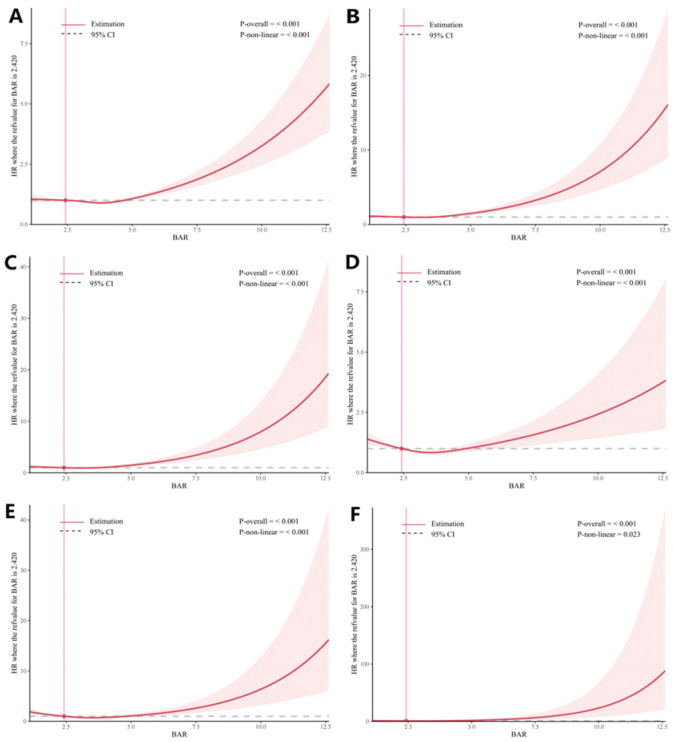
Exposure–response associations between BAR and T2DM transitions using restricted cubic splines with 3 knots. (**A**): pre-T2DM to T2DM; (**B**): T2DM to macrovascular complications; (**C**): T2DM to microvascular complications; (**D**): T2DM to death; (**E**): macrovascular complication to death; (**F**): microvascular complication to death. Model adjusted for age, sex, ethnicity, drinking status, smoking, obese, diet, physical activity, family history of diabetes, hyperlipemia, history of hypertension, and history of cancer, eGFR. Abbreviations: T2DM, type 2 diabetes mellitus; BAR, blood urea nitrogen/albumin.

A nonlinear dose–response relationship was found between BAR and the risk of dynamic progression of T2DM, which indicates the increased risks of dynamic progressions with elevated BAR levels (*p* < 0.05 for all nonlinear results) (Figure 2). Our results revealed that when BAR > 2.42, the risk of diabetes progression increased more significantly with higher BAR levels. Table 2 presents the associations between BUN, albumin, BAR, and the risk of different progressions of T2DM. The crude model showed significant association of BAR with the increased risk of dynamic T2DM progression. The HRs (95% CI) for the dynamic trajectory of T2DM were 1.09 (1.06, 1.12), 1.16 (1.11, 1.22), 1.26 (1.18, 1.34), 1.06 (0.96, 1.16), 1.10 (1.01,1.19), and 1.17 (1.05,1.31). In Model 2, the relationship between BAR and dynamic progressions of T2DM remained significant, and the HRs (95% CI) per BAR increase due to the exposure reached, 1.05 (1.02, 1.09), 1.18 (1.12, 1.25), 1.18 (1.08, 1.28), 0.96 (0.86, 1.08), 0.96 (0.86, 1.08), and 1.18 (1.00, 1.39), except for the route from T2DM or macrovascular complications to death. The associations of BAR with the increased risk of T2DM and related events were notably stronger than those of single BUN levels.

The associations between BAR exposures exhibited positive correlations with the elevated risks of diabetes complications. Moreover, the associations between BAR and the risk of diabetes-related mortality exhibited a stronger connection compared with BAR and all-cause mortality. Table S4 provides detailed insights into the relationships between BAR levels and mortality from specific causes.

### 3.3. Cumulative Transition Probability of T2DM Trajectories

Based on the results presented in Table 2, Figure S2 illustrates the cumulative transition probabilities for the first three progression pathways among individuals aged 60 years throughout the follow-up. Compared with those lowest quartiles of BAR, increases were observed on the cumulative probabilities from pre-T2DM to incident T2DM and subsequent diabetic complications of patients with the highest quartiles of BAR. Participants with a high level of BAR exhibited significantly higher transition probabilities from T2DM to further complications. However, the trajectory from T2DM and post-death did not show significant changes among the participants within the two different levels of BAR.

### 3.4. Sensitivity and Subgroup Analysis

Sensitivity analysis was conducted to evaluate whether cancer, low eGFR (<60 mL/min/1.73 m^2^), extreme BAR values, or a follow-up duration of less than two years confounded the association between BUN and T2DM transitions. Tables S5–S10 show significant and stable associations between BAR and different transitions of T2DM, except for the associations with transition from T2DM to death. Subgroup analysis was performed to confirm the robustness of the correlation between BAR and different stages of T2DM in different subgroups (Figures S3–S8). The connection between BAR and transition from pre-T2DM to T2DM was more pronounced in low physical activity (*p* for interactions <0.001). The association between BAR and transition from T2DM to macrovascular complications were also more pronounced among participants with the history of hypertension. (*p* for interactions = 0.02).

### 3.5. Comparison of Performance Predictions

We conducted analyses of AIC, AUC, and *C-*statistic to evaluate predictive performance. The ability of BAR (AUC: 0.938, *C-*statistic: 0.739, *p* < 0.001) to predict diabetes progression was inferior to that of HbA1c (AUC: 0.949, *C-*statistic: 0.809, *p* < 0.001), and the difference in AIC is greater than 10. The prediction results of the BAR + HbA1c model (AUC: 0.948, *C-*statistic: 0.809, *p* < 0.001) are similar to those of HbA1c alone, but the AIC value of the combined model is the smallest among all. Moreover, BAR was only marginally better than BUN alone (AUC: 0.937, *C-*statistic: 0.738, *p* < 0.001) and was similar to albumin (AUC: 0.940, *C-*statistic: 0.738, *p* < 0.001). Additionally, the AIC value for BUN was higher than those for BAR and albumin, with differences exceeding 10. The specific results are presented in Table S11.

## 4. Discussion

Although this large prospective cohort study found a significant positive correlation between BAR levels and the risk of T2DM progression, particularly among patients with low physical activity or hypertension, its predictive power was inferior to that of HbA1c and only superior to that of BUN, being similar to that of albumin.

Our findings align with experimental and epidemiologic evidence, in that we discovered the association of a high concentration of BUN or low serum levels of albumin with an increased risk of T2DM [16,28,32]. In contrast to prior studies confined to specific populations, such as men [28], pregnant women [37], or patients with diabetic ketoacidosis [38], our findings may provide a compelling case for establishing the association between BAR and T2DM within the broader pre-T2DM. We also found a significant association between high BAR levels and both major and minor complications of diabetes. The obtained results are representative and may be used to bridge the current gap in comprehending the relationship among BAR and subsequent events in patients with T2DM.

BAR is considered as a key risk factor for mortality among critically ill patients [20,23,31], but it cannot reflect an association with dynamic progression. Our findings prove the association of BAR with both the incidence and progression of T2DM, rather than just one disease state. Thus, BAR may contribute to the long-term development of T2DM and the mortality risk of current diseases. Earlier studies have demonstrated that elevated BAR levels are positively associated with all-cause mortality among patients undergoing hemodialysis [39], ICU patients with sepsis [40], or individuals with diabetic ketoacidosis [38]. The present study revealed positive associations between BAR and transition stages from T2DM to diabetic complications mortality. However, no significant associations were noted for the transition from diabetes or macrovascular complications to all-cause mortality. This finding may provide additional supporting evidence given that BAR may add the risk of fatal events in individuals with T2DM complications due to the intricate BUN cycle [41]. The disparities in our findings can stem from several considerations. Firstly, prior studies primarily examined the transition from a healthy state to diabetes mortality, which may inadequately capture potential variations in the effects of BAR on dynamic transitions. Secondly, although diabetes is identified as a major contributor to mortality, advancements in longevity suggest that most individuals with diabetes may succumb primarily due to diabetes complications [42]. Existing studies often gathered diabetes-related mortality based on the ICD 10 code for diabetes and potentially overlook the premature mortality that is attributable to diabetes-related complications. Moreover, previous studies focused on acute and critical illnesses, while our study examined diabetes, a chronic condition with a prolonged course. Long-term medication use in diabetes may influence BAR levels and their impact on mortality.

The subgroup analysis shows that the association between BAR and the transition from pre-T2DM to T2DM was more pronounced among patients with low physical activity. The protective role of physical activity on diabetes-related events is biologically reasonable. Physical activity can mitigate the risk of vascular events in T2DM by improving factors such as excess body weight, elevated blood pressure, and high lipid levels. Moreover, substantial literature shows that activity supports diabetes management by enhancing beta-cell function, improving insulin sensitivity, and reducing inflammation [43,44]. The association between BAR and the transition from T2DM to macrovascular complications was more evident among patients with hypertension. The primary pathophysiological mechanism is that hypertension can lead to arterial stiffness and rising arterial pulse pressure, and pulsatile shear can impair endothelial function and disrupt metabolism [45]. Furthermore, arterial stiffness may result in capillary diastolic dysfunction, leading to a decline in tissue perfusion [46]. This suggests the importance of adopting a multifaceted approach to diabetes prevention and management, focusing on halting the progression to more severe complications. Strategies include managing comorbidities effectively and incorporating regular physical activity.

The exact pathological mechanism linking BAR and diabetes remains unclear. Given that BAR has primarily been studied in acute conditions, it is challenging to establish a connection with chronic diseases like diabetes based on previous research. The link between BAR and diabetes may be explained by the characteristics of its two components. Insulin secretory defects are linked to elevated BUN, a main uremic metabolite [47]. Therefore, BUN or other uremic components in kidney disease may accelerate the risk of T2DM [37]. The primary mechanism is that BUN causes the increase in oxidative stress, insulin resistance, and glucose dysregulation [16]. Additionally, elevated BUN levels indicate accumulated hemodynamic and neurohormonal shifts, leading to reduced renal perfusion and stimulation of the renin–angiotensin–aldosterone pathway. Higher catecholamine release and endothelin levels may promote arteriolar vasoconstriction, resulting in detrimental cardiovascular effects [48,49]. Albumin is a critical component of the BAR index and has been shown to correlate strongly with poor state in various chronic diseases [20]. Several mechanisms may explain the link between albumin levels and the incidence of diabetes and its complications. Physiologically, albumin contributes to osmotic balance, capillary function, and ligand transport, and has antioxidant and free radical scavenging properties [50]. Additionally, albumin possesses the ability to reduce inflammation [51]. Moreover, albumin and its ligands induce inflammation and fibrosis, which contribute to renal function loss. Increased albumin filtration leads to excessive tubular reabsorption, causing inflammation, while tubular dysfunction can result in albuminuria. As a result, serum albumin deficiency may increase oxidative stress and inflammation, which are major contributors to diabetes and its complications [32,52]. Recently, BAR has emerged as a biomarker for assessing disease prognosis and is a key prognostic factor for mortality in conditions such as lung cancer and pneumonia. [18,19,53,54]). However, the ability of BAR to predict the progression of diabetes in our study was inferior to the existing gold standard, HbA1c, and did not demonstrate the significant predictive power independent of BUN and albumin. This could be attributed to the fact that BUN and albumin both are influenced by factors such as aging, protein intake, hemorrhage, catabolic conditions, and others [23]. Additionally, most studies on BAR have focused on acute conditions, where elevated BAR may result from renal reabsorption due to a rapid decrease in circulating volume [22]. In contrast, diabetes and its progression typically occur gradually, with ongoing drug monitoring during this process. This may explain why BAR does not significantly predict the progression of diabetes. The causal relationship between BAR and the adverse outcomes of T2DM, subsequent complications, or mortality remains unclear and warrants further exploration in the form of future clinical and epidemiological studies.

This study has several strengths. Firstly, we minimized selection bias by using a large UKB cohort study. Secondly, for the analysis, we adjusted for confounders and conducted stratified analyses to assess the robustness and heterogeneity of the results. Thirdly, multistate models provide a detailed description of the stages individuals typically experience during their diabetes progression, accounting for the sequence of chronic conditions. These findings can help improve integrated risk and treatment models for diabetes at various stages. The findings of this study should be understood in the context of the following limitations. Firstly, this study used baseline BAR exposure data, and potential changes over time, especially after disease diagnosis, were not considered. Nevertheless, this observational cohort study on the association between BAR and different stages of T2DM cannot establish causality. However, the robustness of the results was maintained after the exclusion of individuals with a follow-up duration of less than two years. This finding suggests that the influence of potential bias was minimized. Randomized controlled trials on regulating drugs of BAR are essential to validate the clinical applicability of these findings. Thirdly, the study mainly included individuals of European Caucasian descent, so the results should be generalized with caution. Finally, the intricate interplay among BAR, chronic kidney disease, and diabetes was clearly evident; however, in our study, after excluding patients with diabetic nephropathy, we provided stable evidence that helped minimize the primary bias.

## 5. Conclusions

Although BAR is significantly positively correlated with the risk of progression at different stages of diabetes, its predictive power was inferior to that of HbA1c and only superior to that of BUN. BAR does not demonstrate sufficient clinical significance in predicting diabetes progression, highlighting the need for further investigation.

## Figures and Tables

**Table 1:** Baseline characteristic of participants in the study.

Characteristics	Overall (*n* = 20,039)	BAR				*p* Value
		Q1: 0.81–2.84 (*n* = 5100)	Q2: 2.84–3.34 (*n* = 5100)	Q3: 3.34–3.93 (*n* = 5100)	Q4: 3.93–19.03 (*n* = 5099)	
Age	58.01 (7.66)	55.13 (7.98)	57.36 (7.71)	58.85 (7.17)	60.71 (6.60)	<0.001
Sex						<0.001
Female	10,999 (53.92)	2896 (56.78)	2786 (54.63)	2728 (53.49)	2589 (50.77)	
Male	9400 (46.08)	2204 (43.22)	2314 (45.37)	2372 (46.51)	2510 (49.23)	
Race						<0.001
White	19,014 (93.21)	4539 (89.00)	4773 (93.58)	4851 (95.12)	4851 (95.14)	
Non-white	1385 (6.79)	561 (11.00)	327 (6.41)	249 (4.88)	248 (4.86)	
Smoking status						<0.001
Never smokers	11,112 (54.47)	2829 (55.47)	2785 (54.61)	2788 (54.67)	2710 (53.15)	
Smokers	7429 (36.42)	1632 (32.00)	1847 (36.22)	1916 (37.57)	2034 (39.89)	
Former smoker	1858 (9.11)	639 (12.51)	468 (9.18)	396 (7.76)	355 (6.96)	
Drinking status						<0.001
Never drinkers	1164 (5.71)	333 (6.53)	271 (5.31)	266 (5.22)	294 (5.77)	
Drinkers	793 (3.89)	206 (4.04)	182 (3.57)	171 (3.35)	234 (4.59)	
Former drinker	18,442 (90.41)	4561 (89.43)	4647 (91.12)	4663 (91.43)	4571 (89.65)	
Family history of diabetes						0.042
No	14,526 (71.21)	3570 (70.00)	3605 (70.69)	3667 (71.90)	3684 (72.25)	
Yes	5873 (28.79)	1530 (30.00)	1495 (29.31)	1433 (28.10)	1415 (27.75)	
Hyperlipemia						<0.001
No	14,812 (72.61)	3877 (76.02)	3763 (73.78)	3728 (73.10)	3444 (67.54)	
Yes	5587 (27.39)	1223 (23.98)	1337 (26.22)	1372 (26.90)	1655 (32.46)	
Hypertension						0.852
No	385 (1.89)	100 (1.96)	93 (1.82)	101 (1.98)	91 (1.78)	
Yes	20,014 (98.11)	5000 (98.04)	5007 (98.18)	4999 (98.02)	5008 (98.22)	
History of cancer						<0.001
No	18,468 (90.53)	4705 (92.25)	4606 (90.31)	4618 (90.55)	4539 (89.02)	
Yes	1931 (9.47)	395 (7.75)	494 (9.69)	482 (9.45)	560 (10.98)	
Obese						<0.001
No	13,006 (63.76)	3346 (65.61)	3274 (64.20)	3294 (64.59)	3092 (60.64)	
Yes	7393 (36.24)	1754 (34.39)	1826 (35.80)	1806 (35.41)	2007 (39.36)	
Healthy diet						0.001
No	13,975 (68.51)	3424 (67.14)	3462 (67.88)	3485 (68.33)	3604 (70.68)	
Yes	6424 (31.49)	1676 (32.86)	1638 (32.12)	1615 (31.67)	1495 (29.32)	
Physical activity						<0.001
low	4381 (21.48)	1158 (22.71)	1118 (21.92)	1068 (20.94)	1037 (20.34)	
moderate	8481 (41.58)	2229 (43.71)	2150 (42.16)	2009 (39.39)	2093 (41.05)	
high	7537 (36.95)	1713 (33.59)	1832 (35.92)	2023 (39.67)	1969 (38.62)	
eGFR (mL/min/1.73 m^2^)	90.77 (32.13)	83.05 (23.67)	87.32 (25.20)	91.10 (26.83)	101.71 (44.98)	<0.001

Abbreviations: BAR, blood urea nitrogen/albumin; eGFR, estimated glomerular filtration rate.

**Table 2:** Associations between BUN, Alb, BAR, and risk of six progressions of T2DM using the multi-state Cox regression model.

Transition	Cases	BUN (HR 95% CI)	Albumin (HR 95% CI)	BAR (HR 95% CI)
Crude model				
Pre-T2DM → T2DM	5621	1.02 (1.01, 1.03)	0.87 (0.79, 0.96)	1.09 (1.06, 1.12)
T2DM → Macrovascular complications	1295	1.03 (1.02, 1.05)	0.71 (0.58, 0.87)	1.16 (1.11, 1.22)
T2DM → Microvascular complications	574	1.05 (1.04, 1.07)	0.64 (0.46, 0.88)	1.26 (1.18, 1.34)
T2DM → Death	1264	1.01 (0.98, 1.03)	0.57 (0.41, 0.81)	1.06 (0.96, 1.16)
Macrovascular complications → Death	350	1.02 (0.99, 1.04)	0.89 (0.59, 1.33)	1.10 (1.01, 1.19)
Microvascular complications → Death	95	1.04 (1.01, 1.07)	0.46 (0.20, 1.04)	1.17 (1.05, 1.31)
Model 1				
Pre-T2DM → T2DM	5621	1.01 (0.99, 1.01)	0.79 (0.72, 0.87)	1.04 (1.01, 1.07)
T2DM → Macrovascular complications	1295	1.02 (1.01, 1.04)	0.64 (0.52, 0.78)	1.13 (1.07, 1.18)
T2DM → Microvascular complications	574	1.05 (1.03, 1.07)	0.62 (0.44, 0.86)	1.25 (1.17, 1.33)
T2DM → Death	1264	0.99 (0.96, 1.01)	0.53 (0.38, 0.76)	0.98 (0.89, 1.08)
Macrovascular complications → Death	350	1.01 (0.98, 1.03)	0.93 (0.62, 1.39)	1.05 (0.95, 1.15)
Microvascular complications → Death	95	1.04 (1.01, 1.07)	0.44 (0.19, 1.01)	1.17 (1.05, 1.31)
Model 2				
Pre-T2DM → T2DM	5621	1.01 (1.00, 1.02)	0.86 (0.78, 0.95)	1.05 (1.02, 1.09)
T2DM → Macrovascular complications	1295	1.03 (1.02, 1.05)	0.66 (0.54, 0.81)	1.18 (1.12, 1.25)
T2DM → Microvascular complications	574	1.03 (1.01, 1.06)	0.59 (0.42, 0.82)	1.18 (1.08, 1.28)
T2DM → Death	1264	0.99 (0.96, 1.02)	0.55 (0.38, 0.78)	0.96 (0.86, 1.08)
Macrovascular complications → Death	350	0.99 (0.96, 1.02)	0.98 (0.66, 1.46)	0.96 (0.86, 1.08)
Microvascular complications → Death	95	1.03 (0.99, 1.02)	0.49 (0.20, 1.16)	1.18 (1.00, 1.39)

Model 1 was adjusted for age, sex. Model 2 was adjusted for age, sex, ethnicity, drinking status, smoking, obese, diet, physical activity, family history of diabetes, hyperlipemia, history of hypertension, and history of cancer, eGFR. Abbreviations: BUN, blood urea nitrogen; T2DM, type 2 diabetes mellitus; BAR, blood urea nitrogen/albumin.

## Data Availability

The data of this study can be requested from the UKB Study (https://biobank.ndph.ox.ac.uk/showcase/, accessed on 30 November 2024).

## Supplementary Materials

Supporting information is available for download at: https://www.mdpi.com/article/10.3390/nu17010113/s1, Table S1: The UDI, definition, and measurements of covariates and outcomes; Table S2: The distribution of average BAR exposures among 200,399 individuals; Table S3: Participant characteristics for inclusion and exclusion in the study; Table S4: Link between BAR and risk of mortality from specific causes; Table S5: Sensitivity analysis results after excluding cancer (*n* = 1931); Table S6: Sensitivity analysis results after excluding eGFR (<60 mL/min/1.73 m^2^) < 60 (*n* = 2512); Table S7: Sensitivity analysis results after excluding the maximum (>99%) or minimum (1%) value of BAR (*n* = 398); Table S8: Sensitivity analysis results after excluding follow-up less than two years (*n* = 1176); Table S9: Sensitivity analysis results after excluding the patients with both macrovascular and microvascular complications (*n* = 198); Table S10: Associations between BAR and risk of four progressions of T2DM (patients with diabetic pan-vascular disease); Table S11: Using the AIC, AUC, and *C-*statistic to compare the predictive performance of multi-state Cox models for the association of BUN, BAR, and albumin with diabetes progression. Figure S1: Follow chart; Figure S2: Transition probabilities for T2DM in 60-year-old participants across different BAR levels; Figure S3: Association between BAR and transitions from pre-T2DM to T2DM in subgroup analyses; Figure S4: Association between BAR and transitions from T2DM to macrovascular complications in subgroup analyses; Figure S5: Association between BAR and transitions from T2DM to microvascular complications in subgroup analyses; Figure S6: Association between BAR and transitions from T2DM to death in subgroup analyses; Figure S7: Association between BAR and transitions from macrovascular complications to death in subgroup analyses; Figure S8: Association between BUN and transitions from microvascular complications to death in subgroup analyses.

## References

[1] Skou S.T., Mair F.S., Fortin M., Guthrie B., Nunes B.P., Miranda J.J., Boyd C.M., Pati S., Mtenga S., Smith S.M. (2022). Multimorbidity. Nat. Rev. Dis. Primers.

[2] Arnold S.V., Khunti K., Tang F., Chen H., Cid-Ruzafa J., Cooper A., Fenici P., Gomes M.B., Hammar N., Ji L. (2022). Incidence rates and predictors of microvascular and macrovascular complications in patients with type 2 diabetes: Results from the longitudinal global discover study. Am. Heart J..

[3] Joseph P., Leong D., McKee M., Anand S.S., Schwalm J.D., Teo K., Mente A., Yusuf S. (2017). Reducing the Global Burden of Cardiovascular Disease, Part 1: The Epidemiology and Risk Factors. Circ. Res..

[4] Roth G.A., Forouzanfar M.H., Moran A.E., Barber R., Nguyen G., Feigin V.L., Naghavi M., Mensah G.A., Murray C.J. (2015). Demographic and epidemiologic drivers of global cardiovascular mortality. N. Engl. J. Med..

[5] Dal Canto E., Ceriello A., Rydén L., Ferrini M., Hansen T.B., Schnell O., Standl E., Beulens J.W. (2019). Diabetes as a cardiovascular risk factor: An overview of global trends of macro and micro vascular complications. Eur. J. Prev. Cardiol..

[6] Geng T., Zhu K., Lu Q., Wan Z., Chen X., Liu L., Pan A., Liu G. (2023). Healthy lifestyle behaviors, mediating biomarkers, and risk of microvascular complications among individuals with type 2 diabetes: A cohort study. PLoS Med..

[7] Gupta S., Dominguez M., Golestaneh L. (2023). Diabetic Kidney Disease: An Update. Med. Clin. N. Am..

[8] Feldman E.L., Callaghan B.C., Pop-Busui R., Zochodne D.W., Wright D.E., Bennett D.L., Bril V., Russell J.W., Viswanathan V. (2019). Diabetic neuropathy. Nat. Rev. Dis. Primers.

[9] Rawshani A., Rawshani A., Franzén S., Eliasson B., Svensson A.M., Miftaraj M., McGuire D.K., Sattar N., Rosengren A., Gudbjörnsdottir S. (2017). Mortality and Cardiovascular Disease in Type 1 and Type 2 Diabetes. N. Engl. J. Med..

[10] Bommer C., Heesemann E., Sagalova V., Manne-Goehler J., Atun R., Bärnighausen T., Vollmer S. (2017). The global economic burden of diabetes in adults aged 20–79 years: A cost-of-illness study. Lancet Diabetes Endocrinol..

[11] GBD 2019 Diseases and Injuries Collaborators (2020). Global burden of 369 diseases and injuries in 204 countries and territories, 1990–2019: A systematic analysis for the Global Burden of Disease Study 2019. Lancet.

[12] Arihan O., Wernly B., Lichtenauer M., Franz M., Kabisch B., Muessig J., Masyuk M., Lauten A., Schulze P.C., Hoppe U.C. (2018). Blood Urea Nitrogen (BUN) is independently associated with mortality in critically ill patients admitted to ICU. PLoS ONE.

[13] Zhu Y., Sasmita B.R., Hu X., Xue Y., Gan H., Xiang Z., Jiang Y., Huang B., Luo S. (2022). Blood Urea Nitrogen for Short-Term Prognosis in Patients with Cardiogenic Shock Complicating Acute Myocardial Infarction. Int. J. Clin. Pract..

[14] Ren X., Qu W., Zhang L., Liu M., Gao X., Gao Y., Cheng X., Xu W., Liu Y. (2018). Role of blood urea nitrogen in predicting the post-discharge prognosis in elderly patients with acute decompensated heart failure. Sci. Rep..

[15] Koppe L., Nyam E., Vivot K., Manning Fox J.E., Dai X.Q., Nguyen B.N., Trudel D., Attané C., Moullé V.S., MacDonald P.E. (2016). Urea impairs β cell glycolysis and insulin secretion in chronic kidney disease. J. Clin. Investig..

[16] D’Apolito M., Du X., Zong H., Catucci A., Maiuri L., Trivisano T., Pettoello-Mantovani M., Campanozzi A., Raia V., Pessin J.E. (2010). Urea-induced ROS generation causes insulin resistance in mice with chronic renal failure. J. Clin. Investig..

[17] Zhang L., Xing M., Yu Q., Li Z., Tong Y., Li W. (2024). Blood urea nitrogen to serum albumin ratio: A novel mortality indicator in intensive care unit patients with coronary heart disease. Sci. Rep..

[18] Cui K., Feng S., Mao Y., Luo H., Yang J., Xu R., Bai L. (2024). The association between blood urea nitrogen to albumin ratio and the 28 day mortality in tuberculosis patients complicated by sepsis. Sci. Rep..

[19] Zeng Z., Ke X., Gong S., Huang X., Liu Q., Huang X., Cheng J., Li Y., Wei L. (2022). Blood urea nitrogen to serum albumin ratio: A good predictor of in-hospital and 90-day all-cause mortality in patients with acute exacerbations of chronic obstructive pulmonary disease. BMC Pulm. Med..

[20] Lin Z., Zhao Y., Xiao L., Qi C., Chen Q., Li Y. (2022). Blood urea nitrogen to serum albumin ratio as a new prognostic indicator in critical patients with chronic heart failure. ESC Heart Fail..

[21] Aronson D., Hammerman H., Beyar R., Yalonetsky S., Kapeliovich M., Markiewicz W., Goldberg A. (2008). Serum blood urea nitrogen and long-term mortality in acute ST-elevation myocardial infarction. Int. J. Cardiol..

[22] Milas G.P., Issaris V., Papavasileiou V. (2022). Blood urea nitrogen to albumin ratio as a predictive factor for pneumonia: A meta-analysis. Respir. Med. Res..

[23] Nam K.W., Kwon H.M., Jeong H.Y., Park J.H., Min K. (2024). Blood urea nitrogen to albumin ratio is associated with cerebral small vessel diseases. Sci. Rep..

[24] Bhatia K., Mohanty S., Tripathi B.K., Gupta B., Mittal M.K. (2015). Predictors of early neurological deterioration in patients with acute ischaemic stroke with special reference to blood urea nitrogen (BUN)/creatinine ratio & urine specific gravity. Indian J. Med. Res..

[25] Hong C., Zhu H., Zhou X., Zhai X., Li S., Ma W., Liu K., Shirai K., Sheerah H.A., Cao J. (2023). Association of Blood Urea Nitrogen with Cardiovascular Diseases and All-Cause Mortality in USA Adults: Results from NHANES 1999–2006. Nutrients.

[26] Wu Q., Zheng J., Lin J., Xie L., Tang M., Ke M., Chen L. (2024). Preoperative blood urea nitrogen-to-serum albumin ratio for prediction of in-hospital mortality in patients who underwent emergency surgery for acute type A aortic dissection. Hypertens. Res..

[27] Zhong J.B., Yao Y.F., Zeng G.Q., Zhang Y., Ye B.K., Dou X.Y., Cai L. (2023). A closer association between blood urea nitrogen and the probability of diabetic retinopathy in patients with shorter type 2 diabetes duration. Sci. Rep..

[28] Xie Y., Bowe B., Li T., Xian H., Yan Y., Al-Aly Z. (2018). Higher blood urea nitrogen is associated with increased risk of incident diabetes mellitus. Kidney Int..

[29] Meira-Machado L., de Uña-Alvarez J., Cadarso-Suárez C., Andersen P.K. (2009). Multi-state models for the analysis of time-to-event data. Stat. Methods Med. Res..

[30] Sudlow C., Gallacher J., Allen N., Beral V., Burton P., Danesh J., Downey P., Elliott P., Green J., Landray M. (2015). UK biobank: An open access resource for identifying the causes of a wide range of complex diseases of middle and old age. PLoS Med..

[31] Liu S., Qiu C., Li W., Li X., Liu F., Hu G. (2024). Blood urea nitrogen to serum albumin ratio as a new prognostic indicator in type 2 diabetes mellitus patients with chronic kidney disease. Sci. Rep..

[32] Cai Y.W., Zhang H.F., Gao J.W., Cai Z.X., Cai J.W., Gao Q.Y., Chen Z.T., Liao G.H., Zeng C.R., Chen N. (2023). Serum albumin and risk of incident diabetes and diabetic microvascular complications in the UK Biobank cohort. Diabetes Metab..

[33] de Wreede L.C., Fiocco M., Putter H. (2010). The mstate package for estimation and prediction in non- and semi-parametric multi-state and competing risks models. Comput. Methods Programs Biomed..

[34] Freisling H., Viallon V., Lennon H., Bagnardi V., Ricci C., Butterworth A.S., Sweeting M., Muller D., Romieu I., Bazelle P. (2020). Lifestyle factors and risk of multimorbidity of cancer and cardiometabolic diseases: A multinational cohort study. BMC Med..

[35] Bjerg L., Hulman A., Carstensen B., Charles M., Jørgensen M.E., Witte D.R. (2018). Development of Microvascular Complications and Effect of Concurrent Risk Factors in Type 1 Diabetes: A Multistate Model From an Observational Clinical Cohort Study. Diabetes Care.

[36] Han Y., Hu Y., Yu C., Guo Y., Pei P., Yang L., Chen Y., Du H., Sun D., Pang Y. (2021). Lifestyle, cardiometabolic disease, and multimorbidity in a prospective Chinese study. Eur. Heart J..

[37] Feng P., Wang G., Yu Q., Zhu W., Zhong C. (2020). First-trimester blood urea nitrogen and risk of gestational diabetes mellitus. J. Cell. Mol. Med..

[38] Chen H., Wang Y., Ji R., Li M. (2024). Association between blood urea nitrogen to serum albumin ratio and in-hospital mortality in critical patients with diabetic ketoacidosis: A retrospective analysis of the eICU database. Front. Endocrinol..

[39] Owen W.F., Lew N.L., Liu Y., Lowrie E.G., Lazarus J.M. (1993). The urea reduction ratio and serum albumin concentration as predictors of mortality in patients undergoing hemodialysis. N. Engl. J. Med..

[40] Wang Y., Gao S., Hong L., Hou T., Liu H., Li M., Yang S., Zhang Y. (2023). Prognostic impact of blood urea nitrogen to albumin ratio on patients with sepsis: A retrospective cohort study. Sci. Rep..

[41] Cao Y.F., Li J., Zhang Z., Liu J., Sun X.Y., Feng X.F., Luo H.H., Yang W., Li S.N., Yang X. (2019). Plasma Levels of Amino Acids Related to Urea Cycle and Risk of Type 2 Diabetes Mellitus in Chinese Adults. Front. Endocrinol..

[42] Saeedi P., Salpea P., Karuranga S., Petersohn I., Malanda B., Gregg E.W., Unwin N., Wild S.H., Williams R. (2020). Mortality attributable to diabetes in 20–79 years old adults, 2019 estimates: Results from the International Diabetes Federation Diabetes Atlas, 9(th) edition. Diabetes Res. Clin. Pract..

[43] Liang Y.Y., He Y., Huang P., Feng H., Li H., Ai S., Du J., Xue H., Liu Y., Zhang J. (2024). Accelerometer-measured physical activity, sedentary behavior, and incidence of macrovascular and microvascular events in individuals with type 2 diabetes mellitus and prediabetes. J. Sport Health Sci..

[44] Valenzuela P.L., Ruilope L.M., Santos-Lozano A., Wilhelm M., Kränkel N., Fiuza-Luces C., Lucia A. (2023). Exercise benefits in cardiovascular diseases: From mechanisms to clinical implementation. Eur. Heart J..

[45] Petrie J.R., Guzik T.J., Touyz R.M. (2018). Diabetes, Hypertension, and Cardiovascular Disease: Clinical Insights and Vascular Mechanisms. Can. J. Cardiol..

[46] Wu Z., Yu S., Zhang H., Guo Z., Zheng Y., Xu Z., Li Z., Liu X., Li X., Chen S. (2022). Combined evaluation of arterial stiffness, glycemic control and hypertension for macrovascular complications in type 2 diabetes. Cardiovasc. Diabetol..

[47] Allison S.J. (2016). Diabetes: Urea inhibits insulin secretion in CKD. Nat. Rev. Nephrol..

[48] Fujii R., Melotti R., Gögele M., Barin L., Ghasemi-Semeskandeh D., Barbieri G., Pramstaller P.P., Pattaro C. (2023). Structural equation modeling (SEM) of kidney function markers and longitudinal CVD risk assessment. PLoS ONE.

[49] Mok Y., Ballew S.H., Matsushita K. (2017). Prognostic Value of Chronic Kidney Disease Measures in Patients with Cardiac Disease. Circ. J..

[50] Fanali G., di Masi A., Trezza V., Marino M., Fasano M., Ascenzi P. (2012). Human serum albumin: From bench to bedside. Mol. Asp. Med..

[51] Sullivan D.H., Roberson P.K., Johnson L.E., Mendiratta P., Bopp M.M., Bishara O. (2007). Association between inflammation-associated cytokines, serum albumins, and mortality in the elderly. J. Am. Med. Dir. Assoc..

[52] Eckart A., Struja T., Kutz A., Baumgartner A., Baumgartner T., Zurfluh S., Neeser O., Huber A., Stanga Z., Mueller B. (2020). Relationship of Nutritional Status, Inflammation, and Serum Albumin Levels During Acute Illness: A Prospective Study. Am. J. Med..

[53] Peng X., Huang Y., Fu H., Zhang Z., He A., Luo R. (2021). Prognostic Value of Blood Urea Nitrogen to Serum Albumin Ratio in Intensive Care Unit Patients with Lung Cancer. Int. J. Gen. Med..

[54] Bae S.J., Kim K., Yun S.J., Lee S.H. (2021). Predictive performance of blood urea nitrogen to serum albumin ratio in elderly patients with gastrointestinal bleeding. Am. J. Emerg. Med..

