# Supplementary material for: Association Between Blood Urea Nitrogen/Albumin and the Incidence as Well as Progression of Type 2 Diabetes
Source: Nutrients. 2024 Dec 30;17(1):113. doi: 10.3390/nu17010113 (PMC11723113; doi:10.3390/nu17010113)
Supplement: Supplementary file 1 [file nutrients-17-00113-s001.zip › nutrients-3373834-supplementary.pdf]

Supplemental Materials

Table S1 The UDI, definition and measurements of covariates and outcomes. 2

Table S2 The distribution of average BAR exposures among 200,399 individuals. 4

Table S3 Participant characteristics for inclusion and exclusion in the study. 5

Table S4 Link between BAR and risk of mortality from specific causes. 8

Table S5 Sensitivity analysis results after excluding cancer(N=1,931). 9

Table S6 Sensitivity analysis results after excluding eGFR(<60ml/min/1.73m^2) <60 (N=2,512). 9

Table S7 Sensitivity analysis results after excluding the maximum (>99%) or minimum (1%) value of BAR (N=398) 10

Table S8 Sensitivity analysis results after excluding the Follow-up was less than two years (N=1,176). 10

Table S9 Sensitivity analysis results after excluding the patients with both macrovascular and microvascular complications (N=198). 11

Table S10 Associations between BAR and risk of four progressions of T2DM (patients with diabetic pan-vascular disease). 11

Table S11 Using the AIC, AUC, and C-statistic to compare the predictive performance of multi-state Cox models for the association of BUN, BAR, and albumin with diabetes progression. 12

Figure S1 Follow chart. 13

Figure S2 Cumulative transition probabilities of T2DM for 60-year-old participants exposed to different levels of BAR. 14

Figure S3 Association between BAR and transitions from pre-T2DM to T2DM in subgroup analyses. 15

Figure S4 Association between BAR and transitions from T2DM to macrovascular complications in subgroup analyses. 16

Figure S5 Association between BARand transitions from T2DM to microvascular complications in subgroup analyses. 17

Figure S6 Association between BAR and transitions from T2DM to death in subgroup analyses. 18

Figure S7 Association between BAR and transitions from macrovascular complications to death in subgroup analyses. 19

Figure S8 Association between BUN and transitions from microvascular complications to death in subgroup analyses... 20

Table S1 The UDI, definition and measurements of covariates and outcomes.

Variables	UDI	Notes
Age	21022	The age of the participant on the day they attended an Initial Assessment Centre.
Sex	31	Sex acquired from central registry at recruitment.
Ethnicity	21000	There are two categories: white and non-white.
Smoking status	20116	The current/past smoking status of the participant. Reference: never.
Drinking status	20117	The current/past drinking status of the participant. Reference: never.
Healthy diet	1349; 1369; 1379; 1389; 1329; 1339; 1458; 1289; 1299; 1309;1319	Healthy diet score was evaluated by red meat intake (<median), fish intake (≥median), vegetable intake (≥median) and fruit intake (≥median). One point was given for each favorable diet factor and the total diet score ranges from 0 to 4; a healthy diet was defined as a diet score ≥ 3. Reference: diet score < 3.
Physical activity	22032	Physical activity was classified according to International Physical Activity Questionnaire (IPAQ). Classification: High, Moderate, Low. Reference: low level.
Obese	23104	BMI more than 30kg/m^2 was defined as obese. Reference: no obesity.
Family history of diabetes	20107; 20110; 20111	Family history included the illness of father, mother, and siblings. Reference: no family history of diabetes.
Hypertension	93; 94; 6177	The participants were classified as hypertension if their systolic blood

		pressure >130mmHg or diastolic blood pressure >90mmHg or ever had medication for blood pressure.<br>Reference: no history of hypertension.
High cholesterol	20002; 6153; 6177	High cholesterol was defined as a self-reported history of high cholesterol or taking medications.<br>Reference: no history of high cholesterol.
Cancer	2453; 20001; 40006	Cancer was extracted from the screening question, self-reported data, and national cancer registries.<br>Reference: no history of cancer.
T2DM	130708; 130709; 30750; 2976; 20003	Cases of T2DM were identified by the International Classification of Disease, Tenth version (ICD-10) code families E11. We also collected cases of diabetes identified through HbA1c ≥48mmol/mol, and participants who ever used insulin or other hypoglycemic drugs before recruitment.
Macrovascular complications	131296; 131298; 131300; 131302; 131304; 131306; 131354; 131360; 131362; 131364; 131366; 131368; 42006; 42010; 42012; 42008; 42000; 6150	Cardiovascular diseases were identified through date of first reported angina pectoris, myocardial infarction, ischaemic heart diseases, heart failure, cerebral infarction, stroke or vascular/heart problems diagnosed by doctor.
Microvascular complications	E113; H360; H280; 20002; 20008; 20009; 6148; E112; E180; E183; E184; E185; N083; 30700; 30510; 30500; E114; G990	Microvascular complication included diabetic eye diseases, diabetic kidney diseases and diabetic neuropathy diseases

Death register	40007; 40000; 40001	Death recodes were acquired from central registry and mapped into four-digit ICD-10 code. Cancer mortality was identified based on codes C00-C99. CVD mortality was identified based on codes I00-I99.

Abbreviations: T2DM, type 2 diabetes mellitus.

Table S2 The distribution of average BAR exposures among 200,399 individuals.

Transition	Mean	Standard deviation	25th percentile	Median	75th percentile	Interquartile range	Minimum	Maximum
The whole stage	3.45	0.94	2.84	3.34	3.94	1.1	0.81	19.03
Pre-T2DM → T2DM	3.51	1.05	2.84	3.36	4.00	1.16	0.83	16.84
T2DM → Macrovascular complications	3.72	1.31	2.96	3.52	4.18	1.22	0.83	16.85
T2DM → Microvascular complications	3.82	1.60	2.92	3.50	4.27	1.35	0.83	16.21
T2DM → Death	3.55	1.10	2.89	3.44	4.06	1.17	0.81	17.83
Macrovascular complications→ Death	3.90	1.82	2.93	3.52	4.35	1.42	1.48	16.85
Microvascular complications→ Death	4.54	2.68	2.98	3.77	5.10	2.12	1.48	16.22

Abbreviations: BAR, blood urea nitrogen/albumin; T2DM, type 2 diabetes mellitus.

Table S3 **Participant characteristics for inclusion and exclusion in the study.**

	Excluded (n = 482,108)	Included (n = 20,399)
Age [years, mean (SD)]	56.47(8.12)	58.01(7.66)
Sex		
Female	262,385(54.42)	10,999(53.92)
Male	219,723(45.58)	9,400(46.08)
Race		
White	453,814(94.13)	19,014(93.21)
Non-white	28,294(5.87)	1,385(6.79)
Smoking status		
Never smokers	265,302(55.03)	11,112(54.47)
Smokers	165,672(34.36)	7,429(36.42)
Former smoker	51,134(10.61)	1,858(9.11)
Drinking status		
Never drinkers	22,799(4.73)	1,164(5.71)
Drinkers	17,313(3.59)	793(3.89)

Former drinker	441,996(91.68)	18,442(90.41)
Family history of diabetes		
No	28,290(23.39)	14,526(71.21)
Yes	92,648(76.61)	5,873(28.79)
Cholesterol history		
No	393,876(81.70)	14,812(72.61)
Yes	88,232(18.30)	5,587(27.39)
History of hypertension		
No	13,311(2.76)	385(1.89)
Yes	468,797(97.24)	20,014(98.11)
History of cancer		
No	440,005(91.27)	18,468(90.53)
Yes	42,103(8.73)	1,931(9.47)
Obese		

No	364,208(75.54)	13,006(63.76)
Yes	117,900(24.46)	7,393(36.24)
Healthy diet		
No	260,646(54.06)	13,975(68.51)
Yes	221,462(45.92)	6,424(31.49)
Physical activity		
low	168,576(34.97)	4,381(21.48)
moderate	157,309(32.63)	8,481(41.58)
high	156,223(32.40)	7,537(36.95)

Table S4 **Link between BAR and risk of mortality from specific causes.**

Transition	Cases	Model 2 HR95%CI	P value
T2DM →Death			
To cancer mortality	856	1.03(0.81,1.29)	0.84
To diabetic complications mortality	7	1.78(1.33,2.39)	<0.001
To CVD mortality	44	1.29(0.40,4.18)	0.67
Macrovascular complications →Death			
To cancer mortality	89	0.99(0.92,1.07)	0.62
To diabetic complications mortality	10	1.00(0.52,1.95)	0.99
To CVD mortality	135	0.90(0.66,1.25)	0.54
Microvascular complications →Death			
To cancer mortality	31	0.71(0.34,1.47)	0.35
To diabetic complications mortality	4	1.79(1.20,2.66)	0.004
To CVD mortality	21	1.27(0.76,2.14)	0.37

Model 1 was fully adjusted for the same covariates as Model 2 in the Table 2.
Abbreviations: T2DM, type 2 diabetes mellitus.

Table S5 **Sensitivity analysis results after excluding cancer(N=1,931).**

Transition	Cases	Model 1<br>HR95%CI	P value
Pre-T2DM → T2DM	5,136	1.05(1.02, 1.09)	0.002
T2DM → Macrovascular complications	1,176	1.18(1.12,1.26)	<0.001
T2DM → Microvascular complications	529	1.18(1.08,1.30)	<0.001
T2DM → Death	1,012	0.99(0.88, 1.24)	0.915
Macrovascular complications→ Death	300	0.94(0.83, 1.07)	0.354
Microvascular complications→ Death	86	1.18(0.99, 1.40)	0.065

Model 1 was fully adjusted for the same covariates as Model 2 in the Table 2.
Abbreviations: T2DM, type 2 diabetes mellitus.

Table S6 Sensitivity analysis results after excluding eGFR(<60ml/min/1.73m^2) <60 (N=2,512).

Transition	Cases	Model 1<br>HR95%CI	P value
Pre-T2DM → T2DM	5,136	1.04(1.01, 1.07)	0.01
T2DM → Macrovascular complications	1,176	1.13(1.08,1.19)	<0.001
T2DM → Microvascular complications	529	1.25(1.16,1.34)	<0.001
T2DM → Death	1,012	1.03(0.92, 1.14)	0.649
Macrovascular complications→ Death	300	1.07(0.98, 1.18)	0.136
Microvascular complications→ Death	86	1.09(0.95, 1.25)	0.199

Model 1 was fully adjusted for the same covariates as Model 2 in the Table 2.
Abbreviations: T2DM, type 2 diabetes mellitus.

Table S7 Sensitivity analysis results after excluding the maximum (>99%) or minimum (1%) value of BAR (N=398)

Transition	Cases	Model 1<br>HR95%CI	P value
Pre-T2DM → T2DM	5,456	1.07(1.03, 1.10)	<0.001
T2DM → Macrovascular complications	1,231	1.17(1.10, 1.25)	<0.001
T2DM → Microvascular complications	538	1.18(1.06,1.31)	0.003
T2DM → Death	1,227	1.03(0.91,1.15)	0.68
Macrovascular complications→ Death	318	0.94(0.82, 1.08)	0.40
Microvascular complications→ Death	81	1.13(0.87, 1.48)	0.36

Model 1 was fully adjusted for the same covariates as Model 2 in the Table 2.

Abbreviations: T2DM, type 2 diabetes mellitus.

Table S8 Sensitivity analysis results after excluding the Follow-up was less than two years (N=1,176).

Transition	Cases	Model 1<br>HR95%CI	P value
Pre-T2DM → T2DM	4,545	1.08(1.04, 1.11)	<0.001
T2DM → Macrovascular complications	978	1.18(1.11,1.26)	<0.001
T2DM → Microvascular complications	433	1.25(1.14,1.38)	<0.001
T2DM → Death	1,073	1.11(0.99, 1.24)	0.07
Macrovascular complications→ Death	247	1.11(0.90, 1.25)	0.07
Microvascular complications→ Death	64	1.12(0.92, 1.35)	0.25

Model 1 was fully adjusted for the same covariates as Model 2 in the Table 2.

Abbreviations: T2DM, type 2 diabetes mellitus.

Table S9 Sensitivity analysis results after excluding the patients with both macrovascular and microvascular complications (N=198).

Transition	Cases	Model 1<br>HR95%CI	P value
Pre-T2DM → T2DM	5,423	1.08(1.05, 1.11)	<0.001
T2DM → Macrovascular complications	1,097	1.22(1.15,1.30)	<0.001
T2DM → Microvascular complications	376	1.17(1.06,1.30)	0.003
T2DM → Death	1,587	1.09(0.98, 1.22)	0.12
Macrovascular complications→ Death	289	1.05(0.93, 1.19)	0.43
Microvascular complications→ Death	34	1.12(0.86, 1.45)	0.42

Model 1 was fully adjusted for the same covariates as Model 2 in the Table 2.
Abbreviations: T2DM, type 2 diabetes mellitus.

Table S10 Associations between BAR and risk of four progressions of T2DM (patients with diabetic pan-vascular disease).

Transition	Cases	Model 1<br>HR95%CI	P value
Pre-T2DM → T2DM	5,621	1.10(1.06, 1.13)	<0.001
T2DM →Complications	1,671	1.22(1.16,1.27)	<0.001
T2DM → Death	1,648	1.08(0.97, 1.20)	0.16
Complications→ Death	384	1.08(0.99, 1.19)	0.09

Model 1 was fully adjusted for the same covariates as Model 2 in the Table 2.
Abbreviations: T2DM, type 2 diabetes mellitus.

Table S11 Using the AIC, AUC, and C-statistic to compare the predictive performance of multi-state Cox models for the association of BUN, BAR, and albumin with diabetes progression.

indicator	AIC	AUC (5-year prediction)	C- statistic	P value
**BAR+ HbA1c**	139367.0	0.948	0.809	<0.001
**HbA1c**	139400.7	0.949	0.809	<0.001
**BAR**	141924.8	0.938	0.739	<0.001
**BUN**	141943.5	0.937	0.738	<0.001
**albumin**	141931.9	0.940	0.738	<0.001

Abbreviations: HbA1c, hemoglobin A1c; BAR, blood urea nitrogen/albumin; BUN, blood urea nitrogen; CI, confidence interval; AIC, akaike information criterion; AUC, area under the curve.

Figure S1 Follow chart.

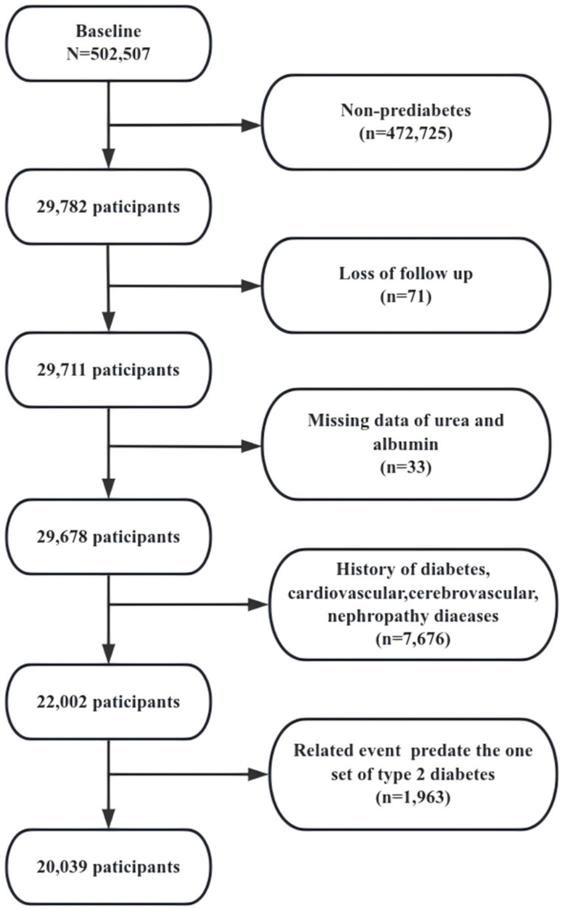

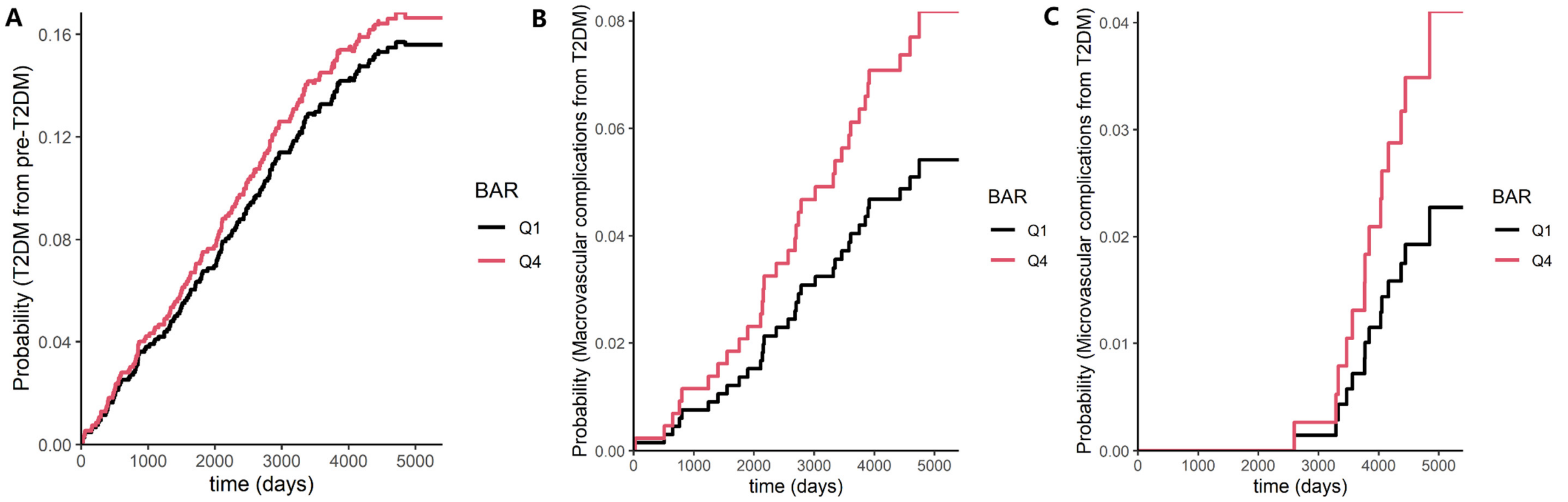

Figure S2 Cumulative transition probabilities of T2DM for 60-year-old participants exposed to different levels of BAR.

Q1: the lowest quartile of BAR concentration (1.09-2.83 mg/g); Q4: above the highest quartile of BAR concentration (3.94-17.83 mg/g). A: pre-T2DM to T2DM; B: T2DM to macrovascular complications; C: T2DM to microvascular complications. Model adjusted for sex, ethnicity, drinking status, smoking status, obese, healthy diet, physical activity, family history of diabetes, history of high cholesterol, history of hypertension and history of cancer, eGFR.

Abbreviations: T2DM, type 2 diabetes mellitus; BAR, blood urea nitrogen/albumin.

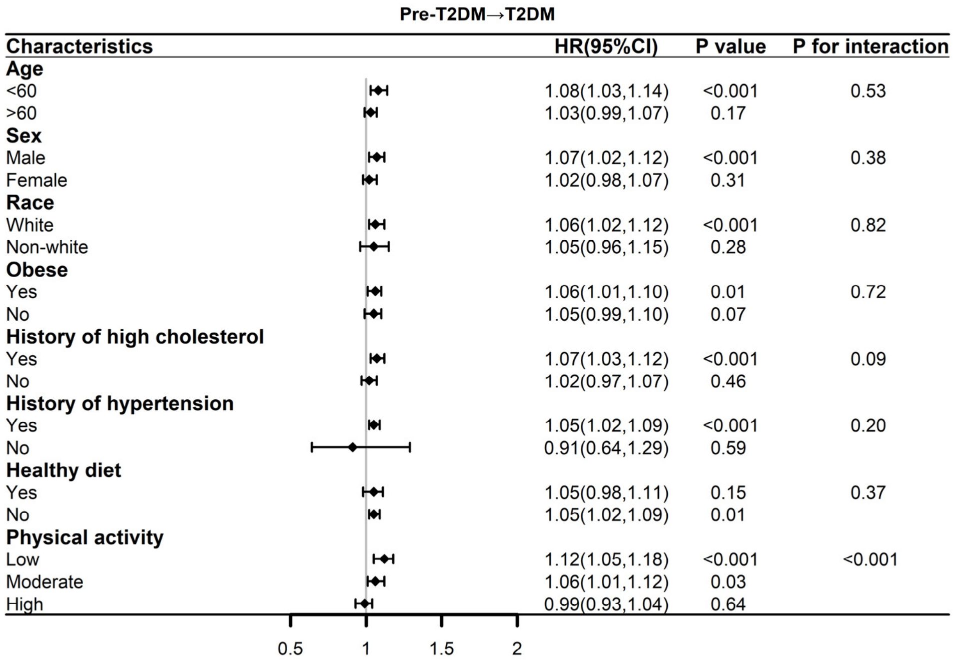

Figure S3 Association between BAR and transitions from pre-T2DM to T2DM in subgroup analyses.

Abbreviations: T2DM, type 2 diabetes mellitus; BAR, blood urea nitrogen/albumin.

T2DM→Macrovascular complications

Characteristics	HR(95%CI)	P value	P for interaction
**Age**			
<60	1.21(1.10,1.33)	<0.001	0.15
>60	1.15(1.07,1.24)	<0.001	
**Sex**			
Male	1.13(1.05,1.22)	<0.001	0.36
Female	1.26(1.14,1.38)	<0.001	
**Race**			
White	1.16(1.10,1.24)	<0.001	0.89
Non-white	1.19(1.02,1.39)	0.03	
**Obese**			
Yes	1.19(1.11,1.28)	<0.001	0.34
No	1.14(1.03,1.26)	0.01	
**History of high cholesterol**			
Yes	1.17(1.08,1.26)	<0.001	0.34
No	1.20(1.09,1.32)	<0.001	
**History of hypertension**			
Yes	1.17(1.10,1.24)	<0.001	0.02
No	3.26(0.64,16.57)	0.16	
**Healthy diet**			
Yes	1.15(1.02,1.29)	0.02	0.46
No	1.19(1.11,1.27)	<0.001	
**Physical activity**			
Low	1.20(1.09,1.31)	<0.001	0.11
Moderate	1.21(1.10,1.34)	<0.001	
High	1.06(0.94,1.19)	0.38	

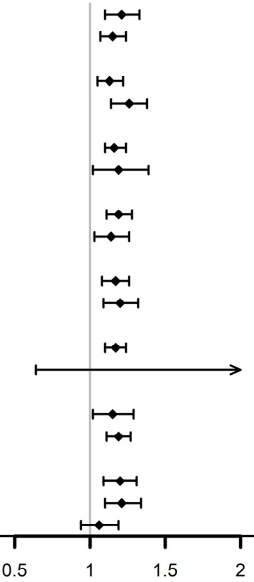

Figure S4 Association between BAR and transitions from T2DM to macrovascular complications in subgroup analyses.

Abbreviations: T2DM, type 2 diabetes mellitus; BAR, blood urea nitrogen/albumi

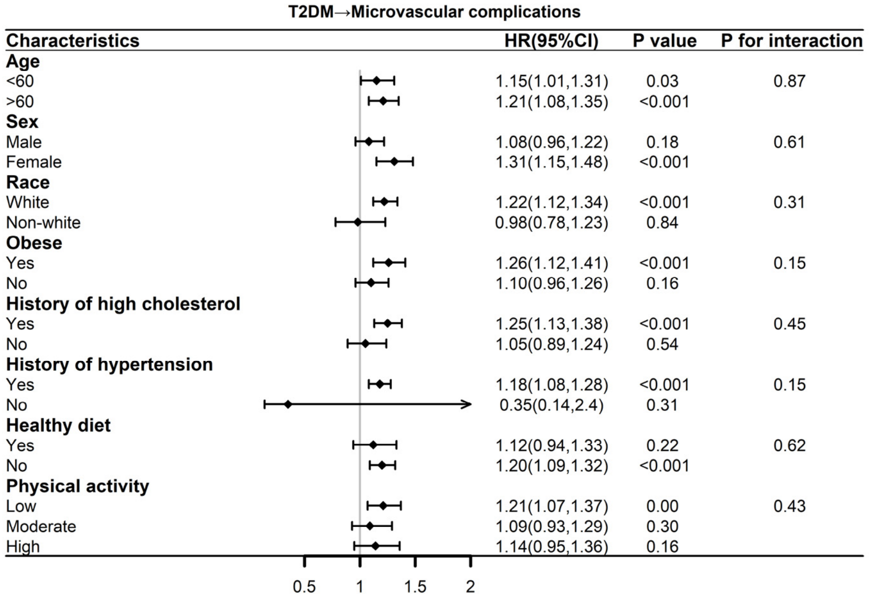

Figure S5 Association between BARand transitions from T2DM to microvascular complications in subgroup analyses.

Abbreviations: T2DM, type 2 diabetes mellitus; BAR, blood urea nitrogen/albumin.

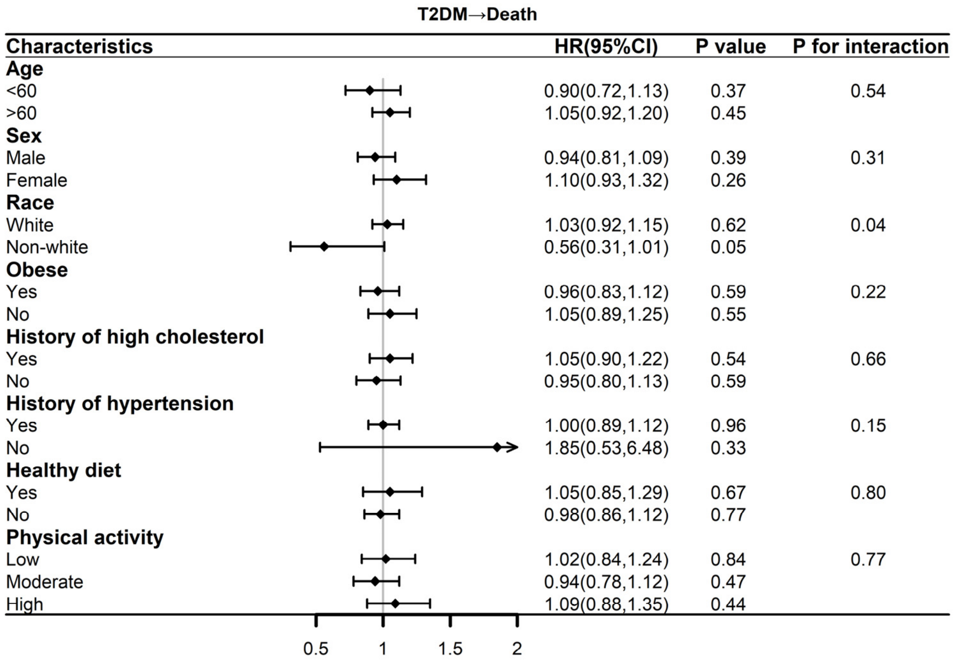

Figure S6 Association between BAR and transitions from T2DM to death in subgroup analyses.

Abbreviations: T2DM, type 2 diabetes mellitus; BAR, blood urea nitrogen/albumin.

.

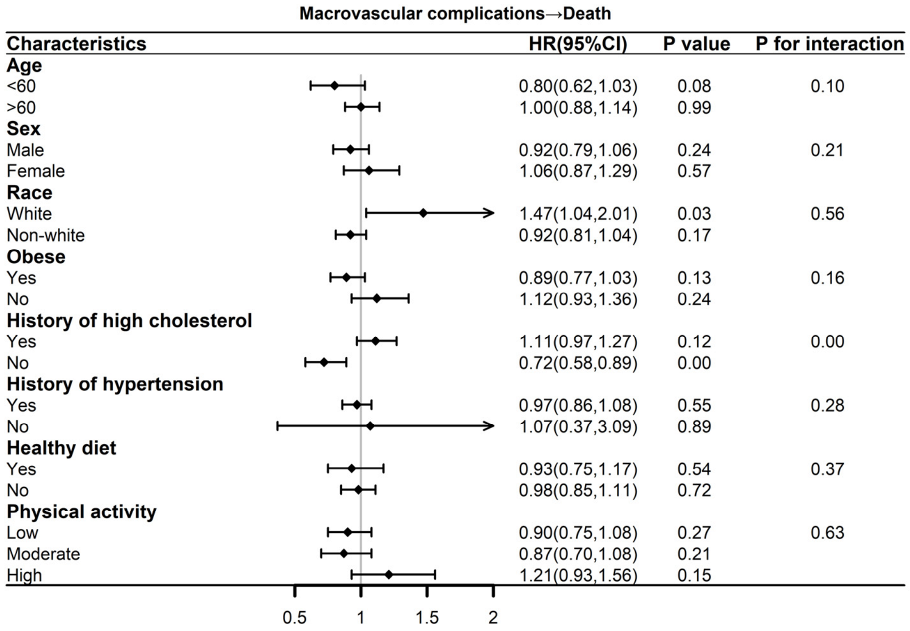

Figure S7 Association between BAR and transitions from macrovascular complications to death in subgroup analyses.

Abbreviations: T2DM, type 2 diabetes mellitus; BAR, blood urea nitrogen/albumin.

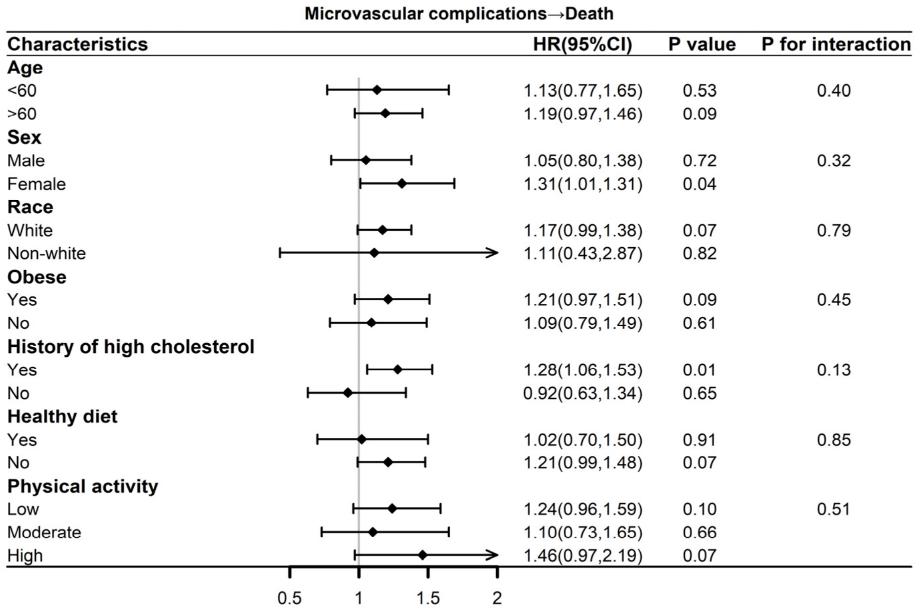

Figure S8 Association between BUN and transitions from microvascular complications to death in subgroup analyses.

Abbreviations: T2DM, type 2 diabetes mellitus; BAR, blood urea nitrogen/albumin.
